# Supplementary material for: A ribosome-associated chaperone enables substrate triage in a cotranslational protein targeting complex
Source: Nat Commun. 2020 Nov 17;11:5840. doi: 10.1038/s41467-020-19548-5 (PMC7673040; doi:10.1038/s41467-020-19548-5)
Supplement: Supplementary file 1 — Supplementary Information [file 41467_2020_19548_MOESM1_ESM.pdf]

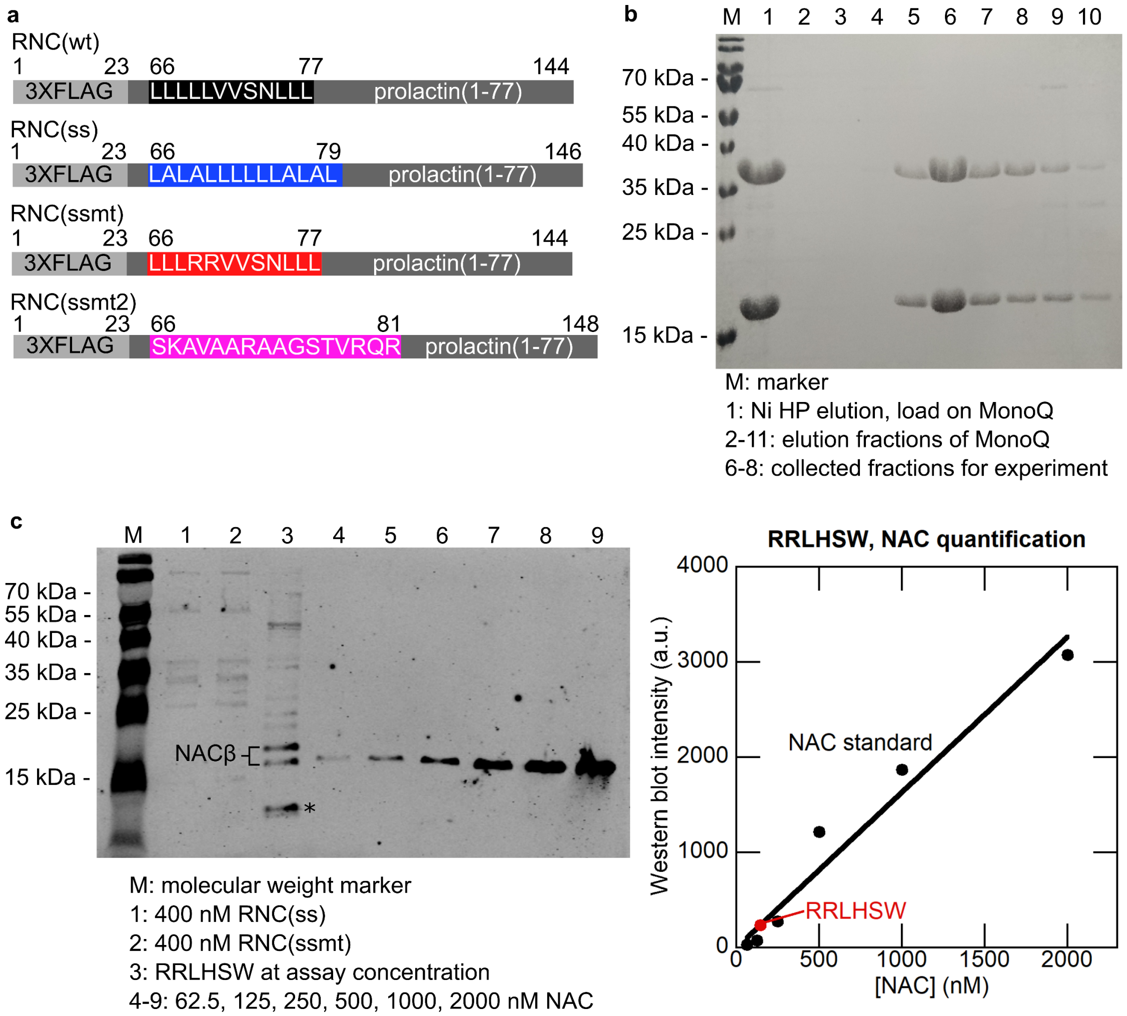

Supplementary Fig. 1: Purification of recombinantly expressed NAC.

a, RNC sequences used in this study.

b, Purification of NAC over the MonoQ ion-exchange column was analyzed by SDS-PAGE and Coomassie staining. NAC containing lanes with the least contamination (lane 6 – 8) were pooled and used for all the experiments. The apparent size of NACα on SDS-PAGE was larger than its predicted molecular weight (23 kDa); this has also been reported by Beatrix et al.[1] The same result has been seen from at least 3 different preparation of NAC.

c, Quantitative western blot to determine the concentration of NAC in HSW(RRL). The left panel shows the western blot with the anti-NACβ antibody. The right panel shows quantification of the NACβ bands. NACβ in mammalian lysate has two splicing isoforms[1], both of which were quantified and gave a total of ~150 nM NAC in the HSW(RRL) derived from 200 nM RRL ribosome. The asterisk indicates a nonspecific band that was not interpreted.

Source data are provided for Figure S1b & S1c.

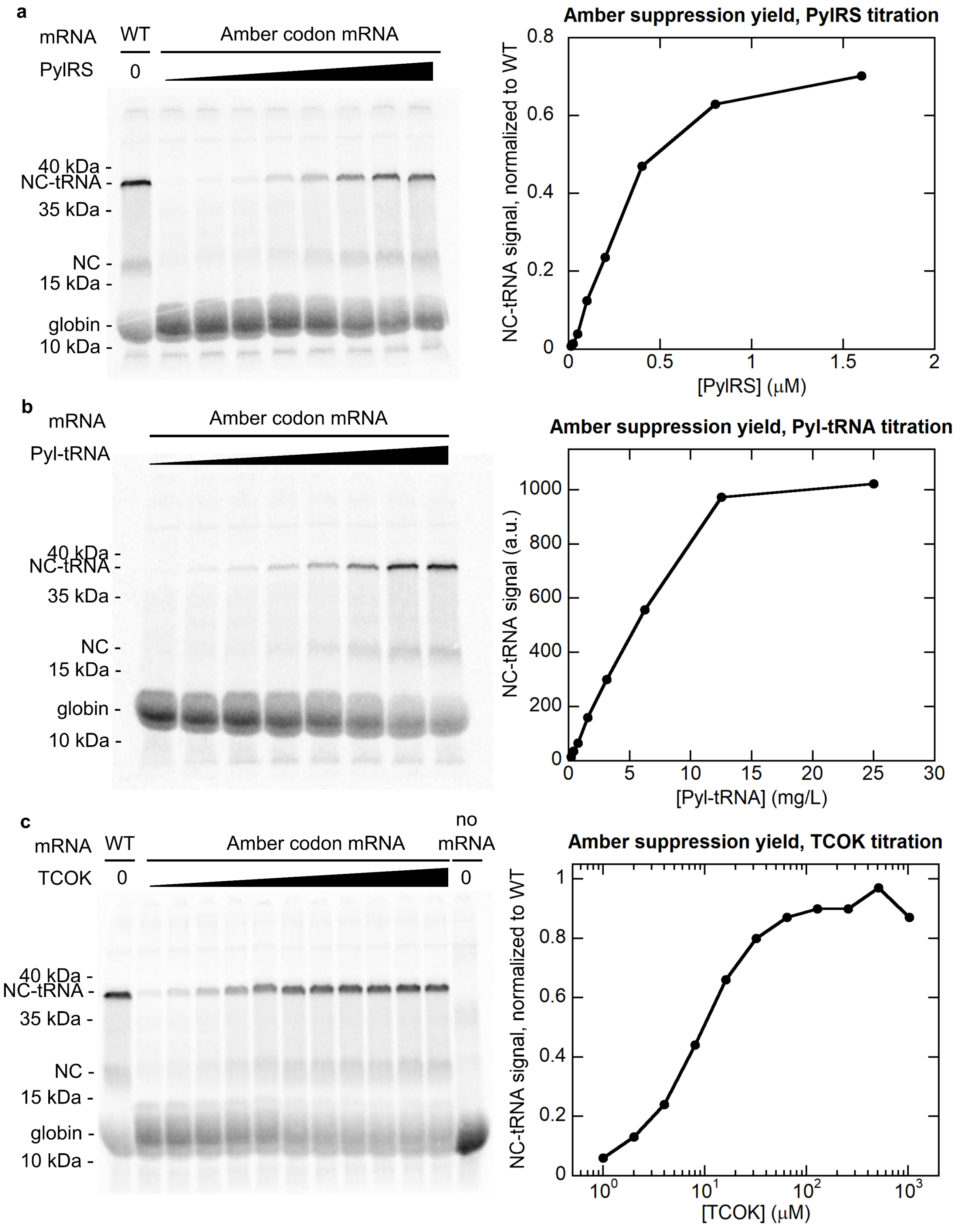

Supplementary Fig. 2: Optimization of amber suppression with *Mm*PyltRNA/RS in RRL.

a, Titration to determine the optimal concentration of *Mm*PylRS. Lane 1 shows RRL in vitro translation of RNC(ss)-encoding mRNA without an amber codon in the absence of *Mm*PylRS,

*Mm*PyltRNA and TCOK. Lanes 2 – 9 show RRL in vitro translation for an mRNA encoding RNC(ss) with an amber codon at the N-terminus of signal sequence. *Mm*PyltRNA and TCOK were present at 20 mg/L and 200 μM, respectively. All translations contained 250 μCi/mL ^{35}S-methionine. Amber suppression yield was quantified from the intensity of the NC-tRNA band normalized to lane 1.

b, Titration to determine the optimal concentration of *Mm*PyltRNA. RRL in vitro translation was carried out similarly to (**a**) but with *Mm*PylRS and TCOK fixed at 1 μM and 200 μM, respectively. Amber suppression yield was quantified from the intensity of the NC-tRNA band.

c, Titration to determine the optimal concentration of TCOK. RRL in vitro translation was carried out similarly to (**a**) but with *Mm*PylRS and *Mm*PyltRNA fixed at 1 μM and 10 mg/L, respectively. The last lane was from translation without added mRNA, *Mm*PylRS, *Mm*PyltRNA, and TCOK. Amber suppression yield was quantified from the intensity of the NC-tRNA band normalized to that in lane 1. The optimal concentrations for *Mm*PylRS (1 μM), *Mm*PyltRNA (10 mg/L) and TCOK (100 μM) were used for all subsequent in vitro translations involving TCOK incorporation.

Source data are provided for Figure S2, a – c.

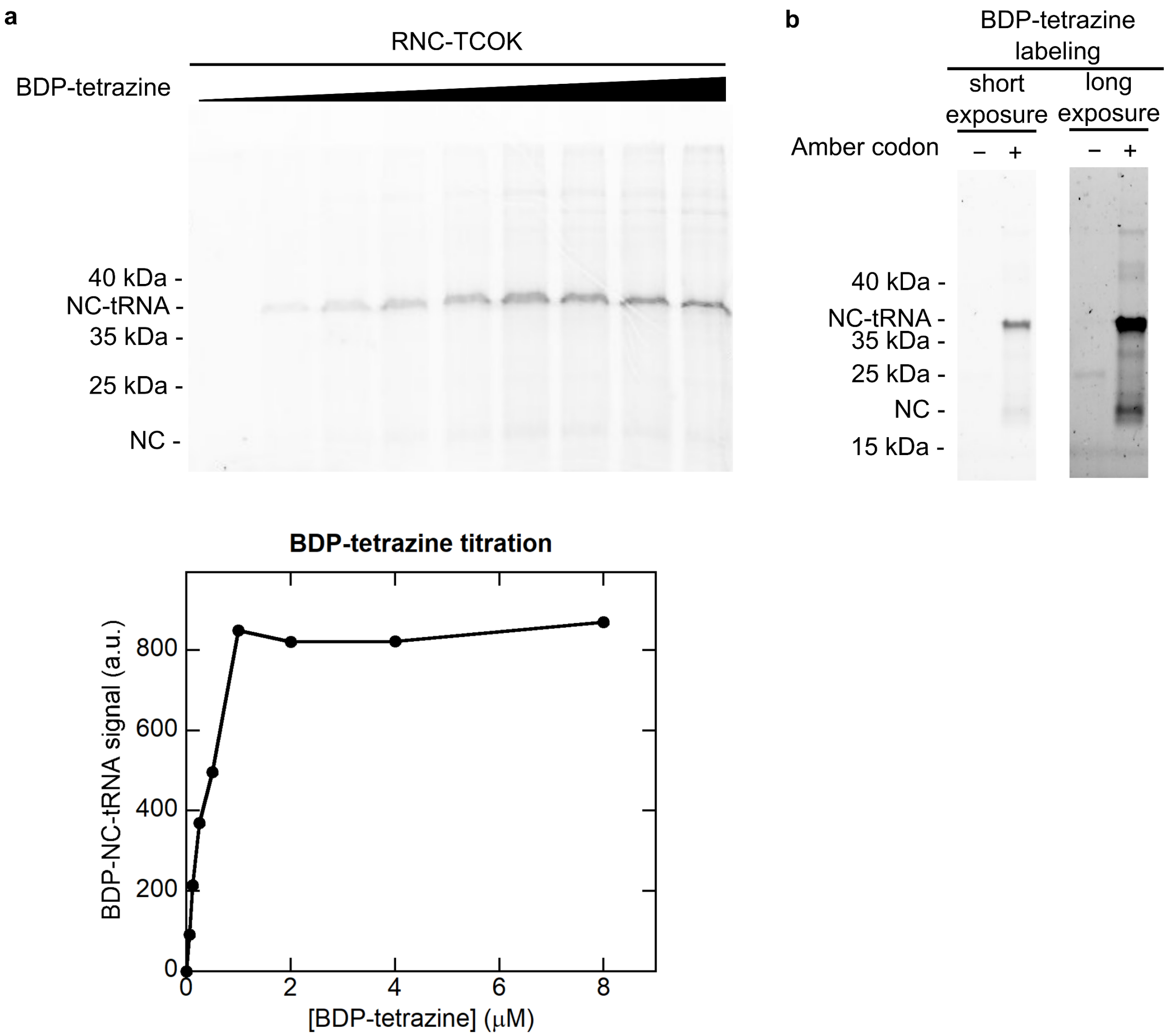

Supplementary Fig. 3: Labeling of RNC with tetrazine-conjugated dye.

a, Optimization of tetrazine-conjugated dye incorporation into the nascent chain. RNC(ss) with TCOK in the nascent chain was purified through a sucrose cushion, resuspended at a final concentration of 1 μM, and incubated with increasing concentrations of tetrazine-conjugated BDP at room temperature for 20 min. The labeling efficiency was quantified using the in-gel fluorescence of BDP. The optimal dye concentration (1 μM) was used for all subsequent labeling.

b, The specificity of tetrazine-based dye labeling. RNC(ss) with or without TCOK incorporated in the nascent chain was labeled under the optimized condition in (**a**). The in-gel fluorescence shows minimal incorporation of dye in the RNC without TCOK incorporation. Similar results have been observed for at least 3 times.

Source data are provided for Figure S3a and S3b.

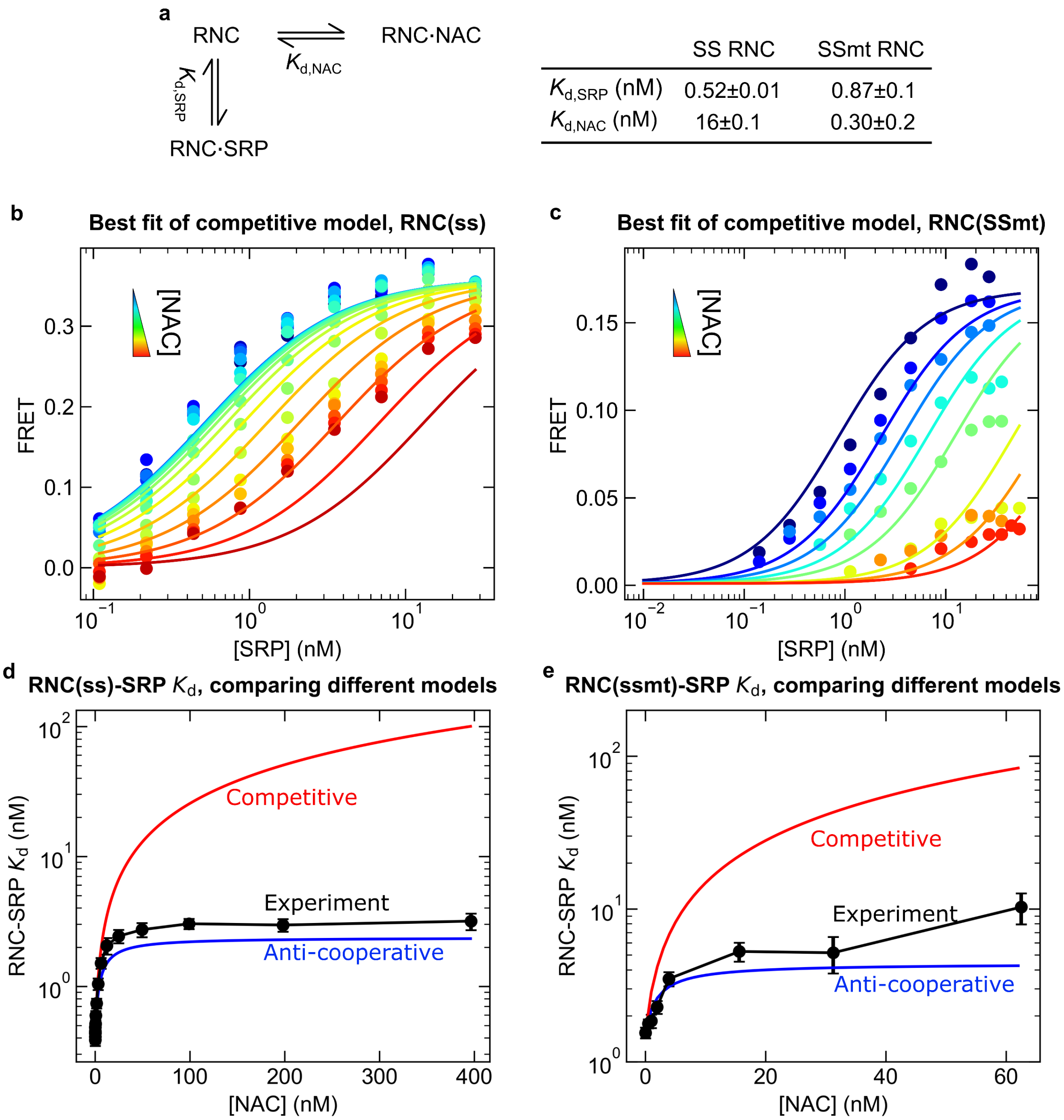

Supplementary Fig. 4: The effect of NAC on RNC-SRP binding cannot be explained by a competitive model

a, Model describing the competitive binding of SRP and NAC to the RNC. The right panel summarizes the best-fit parameters of the model to experimental data, reported as optimized value ± square root of covariance (equivalent to fitting error). The K_d values obtained from this fit differ significantly from those obtained experimentally. For example, $K_{d,NAC}$ for RNC(ss) is 16 nM from this fit compared to 1.6 nM from the measurements in Fig. 4b,c.

b,**c**, Best fits of the RNC-SRP FRET titration data for RNC(ss) (**b**) and RNC(ssmt) (**c**) to the

competitive model in (**a**). The data were from in Fig. 3e,f, and global fitting was done using Eq 7 in the Methods.

d,**e**, The experimentally determined apparent $K_{d,SRP}$ values for RNC(ss) (**d**) and RNC(ssmt) (**e**) were plotted as a function of NAC concentration and compared to predictions from the anti-cooperative model in Fig. 3g (blue lines) and the competitive model in (**a**) (red lines). The anti-cooperative and competitive models were simulated using Equation (12) and (13) in the Methods, respectively, and the $K_{d,SRP}$, $K_{d,NAC}$ and α values summarized in Fig. 3g. The experimental $K_{d,SRP}$ values were from fitting of the individual titrations in Fig. 3e,f to Equation (4) and were shown as fitted value ± fitting error (the square root covariance of the optimized parameters).

Source data are provided for Figures S4d and S4e.

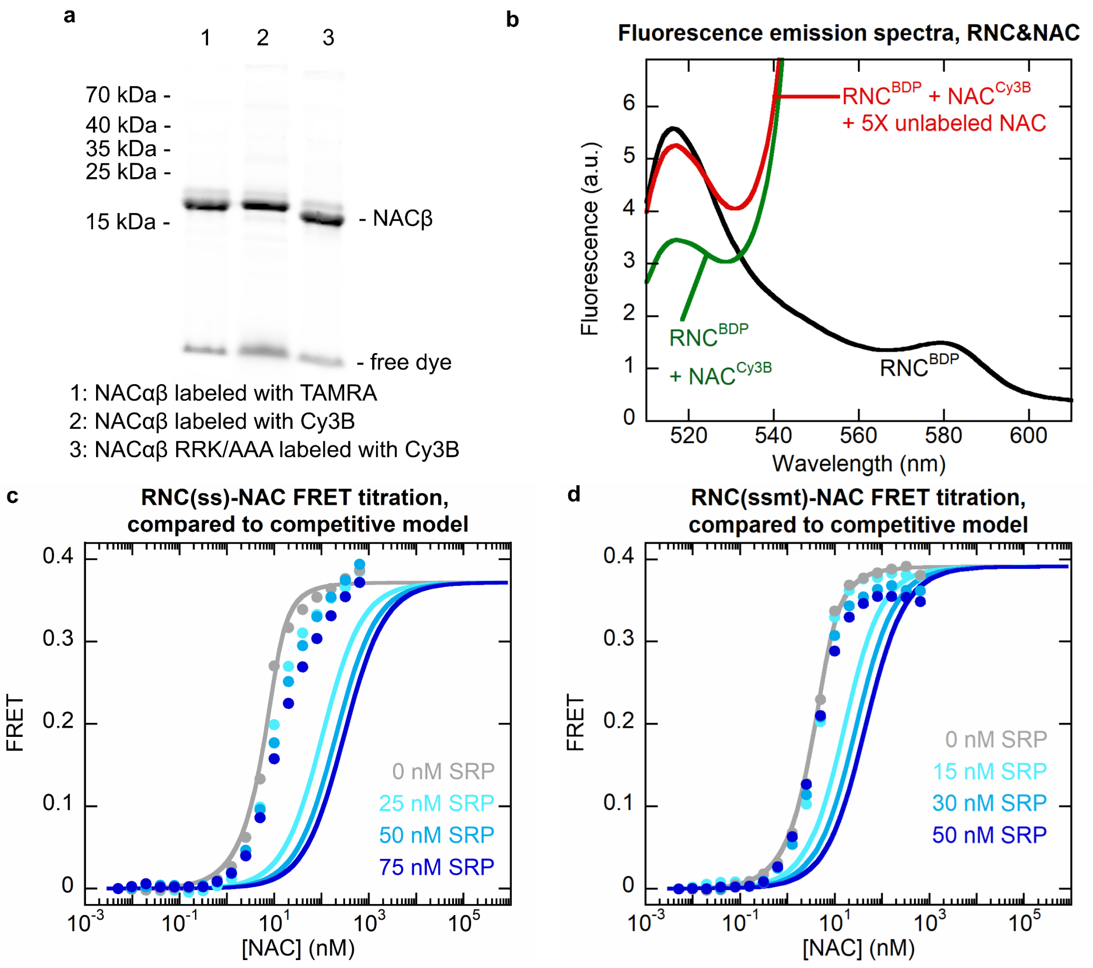

Supplementary Fig. 5: The effect of SRP on RNC-NAC FRET titrations is incompatible with a competitive model

a, Labeling of NAC with maleimide-conjugated acceptor dyes. Wild type NAC and NACmt with a single cysteine at residue 57 of NACβ were labeled with Cy3B or TMR. Labeling of NACβ was visualized by in gel fluorescence. Similar results have been observed for at least 3 times.

b, fluorescence emission spectra showing FRET between RNC(ss)^{BDP} and NAC^{Cy3B}, using an excitation wavelength of 485 nm. Where indicated, the reactions contained 1 nM RNC(ss)^{BDP}, 100 nM NAC^{Cy3B}, and 500 nM unlabeled NAC. **c**,**d**, RNC-NAC FRET titration data (circles) are compared to predictions from the competitive model. The apparent $K_{\mathrm{d,NAC}}$ values predicted by the competitive model in Supplementary Fig. 4a were used to calculate the expected FRET titration curves at the indicated SRP concentrations for RNC(ss) (**b**) and RNC(ssmt) (**c**), respectively, using Equation (5) in the Methods.

Source data are provided for Figures S5a and S5b.

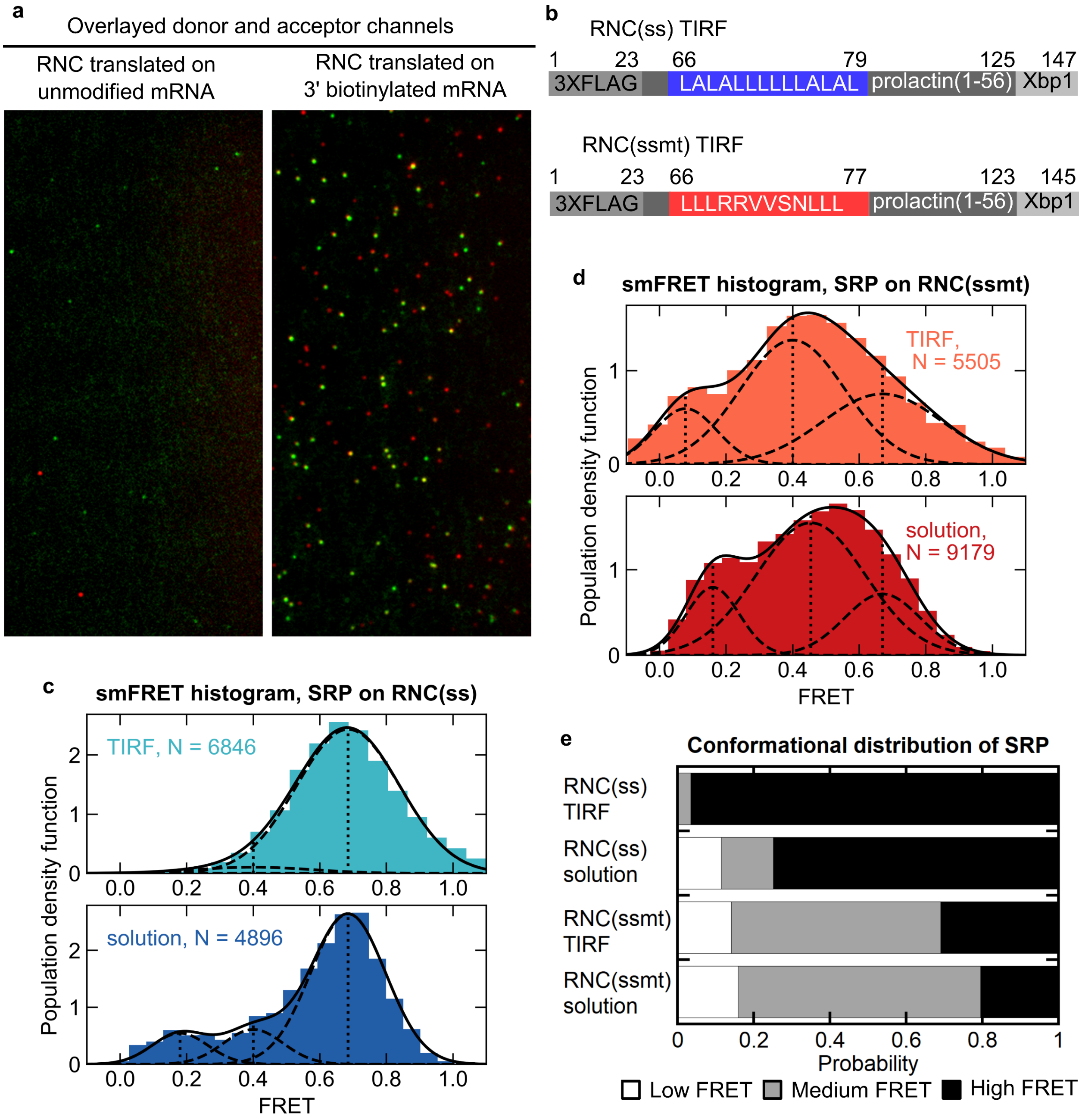

Supplementary Fig. 6: smFRET-TIRF detected conformational distributions of SRP were consistent with the results of solution smFRET measurements.

a, Snapshots of smFRET movies showing specificity for RNC-bound SRP on PEGylated slide. Doubly labeled SRP and RNC with or without mRNA 3'-biotinylation were immobilized as depicted in Fig. 5a and described in the Methods. Images from the donor (green, with donor excitation) and the acceptor (red, with acceptor excitation) channels were aligned to visualize doubly labeled SRP (appear as yellow).

b, Composition of nascent chains on RNC tested with smFRET. The residues in the signal suences are indicated.

c,**d**, Comparison of the smFRET histograms for SRP bound to RNC(ss) (**c**) and RNC(ssmt) (**d**) from the TIRF and solution-based smFRET measurements. The histograms for the TIRF measurements are the same as in Fig. 5**c**,**d**. The histograms for the solution-based measurements were from Lee et al.[2] and shown for comparison. 'N' is the number of frames or number of photon bursts used to construct the histogram for TIRF- and solution-based measurements, respectively. The solid bars are histograms of experimental data, and the solid lines are fits of the data to the sum of three-Gaussian distributions, with the individual Gaussian distributions indicated by dashed lines and the center of each Gaussian function indicated by the vertical dotted lines.

e, Summary of the population distribution of SRP in the low, medium and high FRET states for RNC(ss) and RNC(ssmt) from the TIRF- and solution-based measurements.

Source data are provided for Figure S6e.

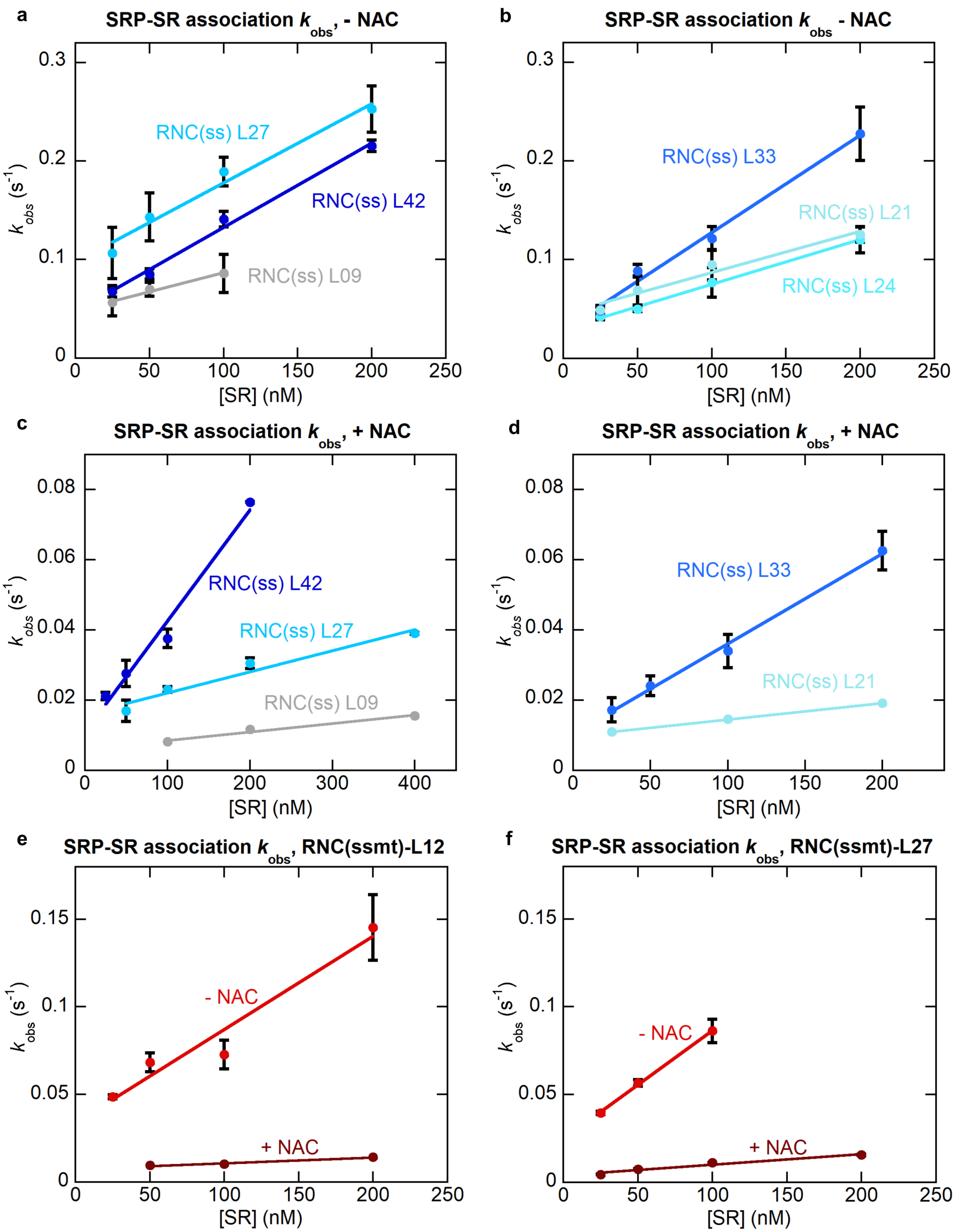
a
SRP-SR association k obs, - NAC
RNC(ss) L27
RNC(ss) L42
RNC(ss) L09
k obs (s-1)
0 0.1 0.2 0.3
0 50 100 150 200 250
[SR] (nM)
b
SRP-SR association k obs - NAC
RNC(ss) L33
RNC(ss) L21
RNC(ss) L24
k obs (s-1)
0 0.1 0.2 0.3
0 50 100 150 200 250
[SR] (nM)
c
SRP-SR association k obs, + NAC
RNC(ss) L42
RNC(ss) L27
RNC(ss) L09
k obs (s-1)
0 0.02 0.04 0.06 0.08
0 100 200 300 400
[SR] (nM)
d
SRP-SR association k obs, + NAC
RNC(ss) L33
RNC(ss) L21
k obs (s-1)
0 0.02 0.04 0.06 0.08
0 50 100 150 200
[SR] (nM)
e
SRP-SR association k obs, RNC(ssmt)-L12
- NAC
+ NAC
k obs (s-1)
0 0.05 0.1 0.15
0 50 100 150 200 250
[SR] (nM)
f
SRP-SR association k obs, RNC(ssmt)-L27
- NAC
+ NAC
k obs (s-1)
0 0.05 0.1 0.15
0 50 100 150 200 250
[SR] (nM)

Supplementary Fig. 7: SRP-SR association kinetics depends on the length of NC.

a,**b**, Apparent SRP-SR association rate constants on RNC(ss) with different NC lengths were measured and plotted as a function of SR concentration. NC lengths are defined by the number of amino acids C-terminal to the signal sequence. All data are shown as mean ± SD, with n = 3 – 5 independent measurements on the same biological sample. Linear fits of the data (Equation (1)) gave the values of $k_{\mathrm{on,SR}}$ at different nascent chain lengths.

c,**d**, Same as in (**a**) and (**b**), but with 300 nM NAC present.

e,**f**, Same as in (**a**) and (**b**), but with RNC(ssmt) in the absence and presence of NAC.

Source data are provided for Figure S7, a – f.

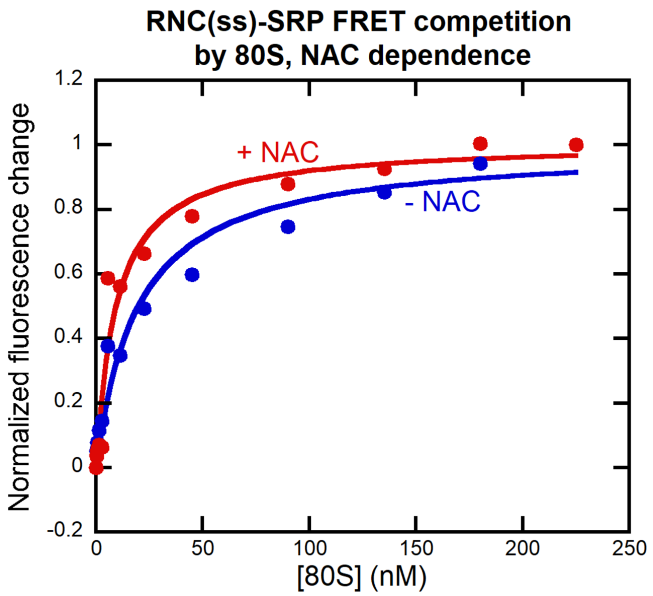

Supplementary Fig. 8: NAC does not weaken the binding of SRP to the 80S ribosome. The binding affinity of SRP for 80S was determined by using 80S as a competitor for RNC-SRP binding in the presence (red) and absence (blue) of 1 uM NAC, as described in the Methods. The lines are fits of the data to Equation (7) in the Methods, which gave K_i values of 19.6 nM and 9.5 nM in the absence and presence of NAC. Source data are provided.

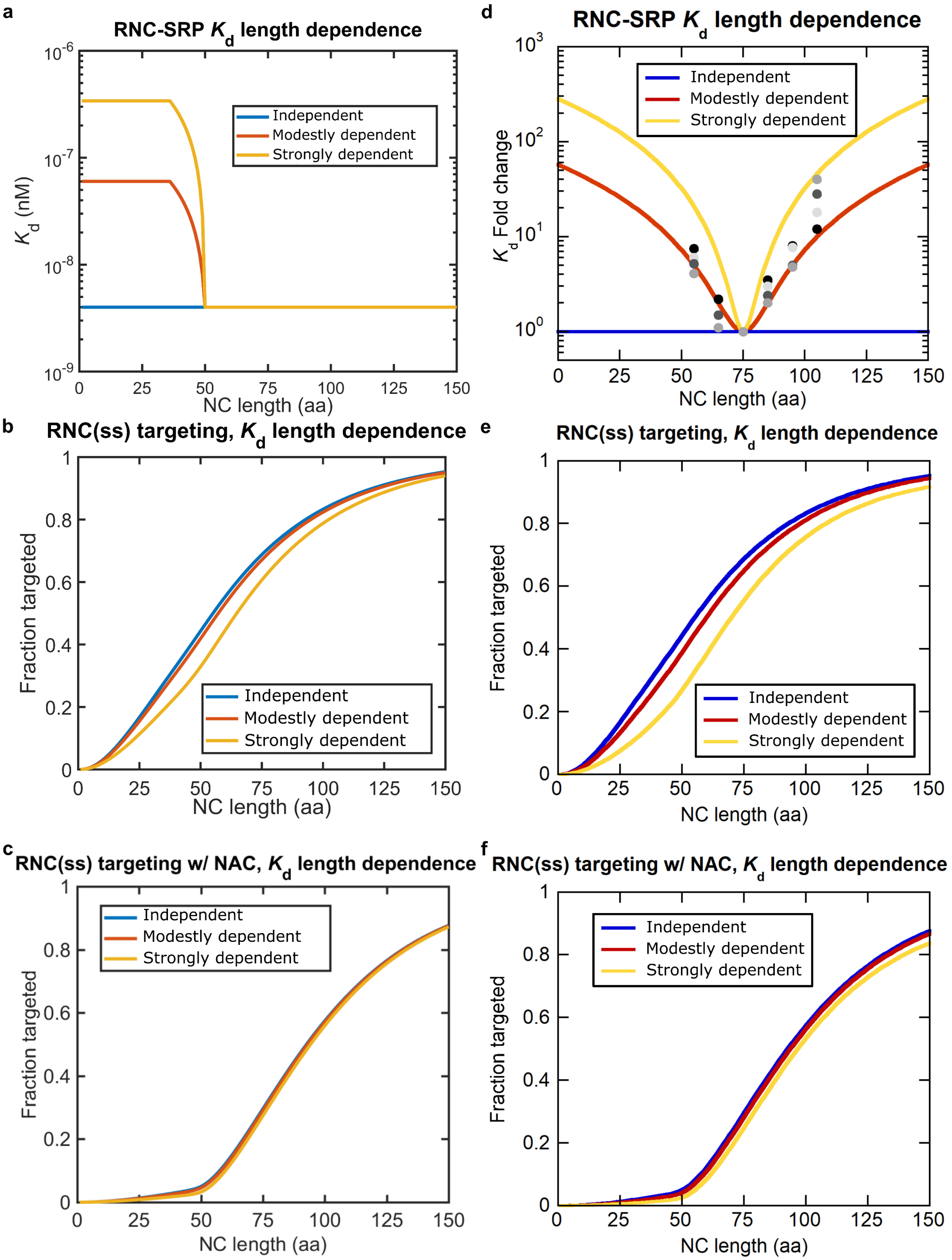

Supplementary Fig. 9: Modeling of cotranslational targeting with length-dependent RNC-SRP K_d.

Modeled progression curves for cotranslational protein targeting using the length-dependent RNC-SRP K_d values specified in (a) for (b) and (c), or (d) for (e) and (f). (d) Experimental results from Figure 5D in Noriega et al.[3] are replotted here (grey and black dots) and then parameterized with

parabolic curves (yellow and red lines) to mimic the trend for modeling. All the other parameters for the modeling are the same as those specified in Fig. 7d.

Source data are provided for Figure S9, a – f.

Supplementary Table 1, primer sequences

Primer name	Sequence
NAC vec F	GATGAGGCTTCCAAGAATGAGGCAAACTAATAACTGGTGCCGCGCGGCAGCCATATGGC
NAC vec R	GGACGGTTTCTGTGGCTTCGCCGGGCATGCCGCTGCTGTGATGATGATGATGATGGCTG
NAC ins1 F	CAGCCATCATCATCATCATCACAGCAGCGGCATGCCCGGCGAAGCCACAGAAACCGTCC
NAC ins1 R	ATATCTCCTTCTTAAAGTTAAACAAAATTATTATTACATTGTTAATTCCATAATCGCATTTACAATATCATTACTGTTGTTCTTCAGGGC
NAC ins2 F	AATAATTTTGTTTAACTTTAAGAAGGAGATATACCATGAAAGAAACAATCATGAACCAGG
NAC ins2 R	GCCATATGGCTGCCGCGCGGCACCAGTTATTAGTTTGCCTCATTCTTGGAAGCCTCATC
NAC RRK/AAA F	GGAACTGCTGCGGCCGCAAAGAAGGTGGTTCATAGAACAGCCACAGCAGATGAC
NAC RRK/AAA R	CACCTTCTTTGCGGCCGCAGCAGTTCCTTTCCCACCAATGCGCACTTGTGCCTGC
MmPylRS vec F	CTGGCTCCGAACCTGGCGAACTACCTGCGTAAACTGGACCGTGCTCTGCCGGACCCGATC
MmPylRS vec R	CATGGTATATCTCCTTCTTAAAGTTAAACAAAATTATTTCTAGAGGGGAATTGTTATCC
MmPylRS ins F	TTTTGTTTAACTTTAAGAAGGAGATATACCATGGACAAAAAACCGCTGAACACCCTGATC
MmPylRS ins R	TTTACGCAGGTAGTTCGCCAGGTTCGGAGCCAGCATCGGACGCAGGCAGAAGTTTTTGTC
MmPyltRNA F	ACGATCAGCATAATACGACTCACTATAGGGAACCTGATCATGTAGATCGAATGGACTCTA
MmPyltRNA R	TGGCGGAAACCCCGGGAATCTAACCCGGCTGAACGGATTTAGAGTCCATTCGATCTACAT

EMCV IRES vec F	ACCATGGACTATAAAGACCATGACGGGGATTAC
EMCV IRES vec R	CCCTATAGTGAGTCGTATTAATTTCGCGGGATCG
EMCV IRES ins F	TAATACGACTCACTATAGGGCCCCCCCCCCCTAACGTTACTGGC
EMCV IRES ins R	GGTCTTTATAGTCCATGGTTGTGGCCATATTATCATCGTGTTTTTCAAAGG
pPL amb F	AAAGGGTCCTAGCTGGCCCTGGCCCTACTGCTACTGCTAC
pPL amb R	CAGGGCCAGCTAGGACCCTTTCTGCGACGAACCTTTGCTG
pPL to ss F	TGCTCCTGCTACTCGCCCTCGCCCTCTGCCAGGGTGTGGTCTCCACCCCCGTCTGTCC
pPL to ss R	GAGGGCGAGTAGCAGGAGCAGCAGGGCCAGGGCCAGGCGGGACCCTTTCTGCGACGAACC
pPL to ssmt F	CTGCGCCGAGTGGTGTCAAATCTACTCTTGTGCCAGGGTGTGG
pPL to ssmt R	CACCACTCGGCGCAGGAGCAGGCGGGACCCTTTCTGCGACGAACC
pPL to ssmt2 F	CCCGGGCGGCGGGATCCACTGTCCGGCAGAGGTGCCAGGGTGTGGTCTCCACCCCCGTC
pPL to ssmt2 R	AGTGGATCCCGCCGCCCGGGCGGCCACTGCTTTGCTGCGGACCCTTTCTGCGACGAACC
mRNA template PCR F	CGGCCAGTGAATTCGAGCTCGG
mRNA template PCR R	CACGGTATGGCAGCTGTTGAGGGCC

References

1. Beatrix, B., Sakai, H. &Wiedmann, M. The α and β subunit of the nascent polypeptide-associated complex have distinct functions. *J. Biol. Chem.* **275**, 37838–37845 (2000).
2. Lee, J. H. *et al.* Sequential activation of human signal recognition particle by the ribosome and signal sequence drives efficient protein targeting. *Proc. Natl. Acad. Sci. U. S. A.* **115**, E5487–E5496 (2018).
3. Noriega, T. R. *et al.* Signal recognition particle-ribosome binding is sensitive to nascent chain length. *J. Biol. Chem.* **289**, 19294–19305 (2014).
