# Supplementary material for: A ribosome-associated chaperone enables substrate triage in a cotranslational protein targeting complex
Source: Nat Commun. 2020 Nov 17;11:5840. doi: 10.1038/s41467-020-19548-5 (PMC7673040; doi:10.1038/s41467-020-19548-5)
Supplement: Supplementary file 3 — Reporting Summary [file 41467_2020_19548_MOESM3_ESM.pdf]

nature research

Corresponding author(s): Shu-ou Shan

Last updated by author(s): Oct 15, 2020

Reporting Summary

Nature Research wishes to improve the reproducibility of the work that we publish. This form provides structure for consistency and transparency in reporting. For further information on Nature Research policies, see our Editorial Policies and the Editorial Policy Checklist.

Statistics

For all statistical analyses, confirm that the following items are present in the figure legend, table legend, main text, or Methods section.

n/a	Confirmed	
☐	☒	The exact sample size (*n*) for each experimental group/condition, given as a discrete number and unit of measurement
☐	☒	A statement on whether measurements were taken from distinct samples or whether the same sample was measured repeatedly
☒	☐	The statistical test(s) used AND whether they are one- or two-sided<br>*Only common tests should be described solely by name; describe more complex techniques in the Methods section.*
☒	☐	A description of all covariates tested
☒	☐	A description of any assumptions or corrections, such as tests of normality and adjustment for multiple comparisons
☐	☒	A full description of the statistical parameters including central tendency (e.g. means) or other basic estimates (e.g. regression coefficient) AND variation (e.g. standard deviation) or associated estimates of uncertainty (e.g. confidence intervals)
☒	☐	For null hypothesis testing, the test statistic (e.g. *F*, *t*, *r*) with confidence intervals, effect sizes, degrees of freedom and *P* value noted<br>*Give P values as exact values whenever suitable.*
☒	☐	For Bayesian analysis, information on the choice of priors and Markov chain Monte Carlo settings
☒	☐	For hierarchical and complex designs, identification of the appropriate level for tests and full reporting of outcomes
☒	☐	Estimates of effect sizes (e.g. Cohen's *d*, Pearson's *r*), indicating how they were calculated

Our web collection on statistics for biologists contains articles on many of the points above.

Software and code

Policy information about availability of computer code

Data collection	micromanager v1.4
Data analysis	Kaleidagraph v4.0, ImageJ v1.52a, Chimera v1.14rc, MATLAB 2017a (9.2.0.538062), iSMS v2.01, custom code for simulation in Fig. 6: https://github.com/emc2emc2/2020_cotranslational_targeting

For manuscripts utilizing custom algorithms or software that are central to the research but not yet described in published literature, software must be made available to editors and reviewers. We strongly encourage code deposition in a community repository (e.g. GitHub). See the Nature Research guidelines for submitting code & software for further information.

Data

Policy information about availability of data

All manuscripts must include a data availability statement. This statement should provide the following information, where applicable:
- Accession codes, unique identifiers, or web links for publicly available datasets
- A list of figures that have associated raw data
- A description of any restrictions on data availability

Data supporting the findings of this manuscript are available from the corresponding author upon reasonable request. Structural data associated with Fig. 1a are available in: ribosome: PDB: 4UG0; SRP: EMD-3037, NAC: EMD-4938; RAC: EMD-6105; and NatA/E: EMD-0202. Source data are provided with this paper.

Field-specific reporting

Please select the one below that is the best fit for your research. If you are not sure, read the appropriate sections before making your selection.

☒ Life sciences ☐ Behavioural & social sciences ☐ Ecological, evolutionary & environmental sciences

For a reference copy of the document with all sections, see nature.com/documents/nr-reporting-summary-flat.pdf

Life sciences study design

All studies must disclose on these points even when the disclosure is negative.

Sample size	Most biochemical measurements were repeated 3 to 5 times to allow statistical analysis and ensure reproducibility, and are specified in figure legends. Sample size for single molecule experiments are specified in the respective data figures.<br><br>Randomization/blinding only apply to cellular/animal/patient/population studies that test null hypothesis, and where measurable differences are small even if statistically significant. These procedures do not apply to biochemical and biophysical studies that measure rate and equilibrium constants of molecular reactions in this study. All the statistics for the biochemical and single molecular measurements in this work were performed according to the standards of the respective field. Moreover, only large differences (more than 10-fold) are considered significant in these measurements, which are well beyond experimental error range.
Data exclusions	No data were excluded.
Replication	The kinetic and fluorescence measurements were repeated 3 to 5 times, and the results were successfully replicated. SDS-PAGE gel data are not repeated because they are extremely clear (Fig. S2, S3 and S5) or show results corroborated by previous studies (Fig. 1, S1).
Randomization	Not applicable, as commented above.
Blinding	Not applicable, as commented above.

Reporting for specific materials, systems and methods

We require information from authors about some types of materials, experimental systems and methods used in many studies. Here, indicate whether each material, system or method listed is relevant to your study. If you are not sure if a list item applies to your research, read the appropriate section before selecting a response.

Materials & experimental systems

n/a	Involved in the study	
☐	☒	Antibodies
☒	☐	Eukaryotic cell lines
☒	☐	Palaeontology and archaeology
☒	☐	Animals and other organisms
☒	☐	Human research participants
☒	☐	Clinical data
☒	☐	Dual use research of concern

Methods

n/a	Involved in the study	
☒	☐	ChIP-seq
☒	☐	Flow cytometry
☒	☐	MRI-based neuroimaging

Antibodies

Antibodies used	anti-NACβ antibody (Abcam) RRID: AB_2783867,<br>IRDye 800CW goat anti–rabbit IgG (LI-COR Biosciences) RRID: AB_2651127
Validation	All antibodies are validated by the manufacturer. WB with Anti-NACβ has been tested by the manufacturer with following cells: MCF7, SK-BR-3, HeLa, Jurkat, C6, RAW 264.7, PC-12 and NIH/3T3 cell lysates; Human lymph node and fetal kidney lysates; Mouse heart and spleen lysates. Anti-NACβ antibody was additionally validated by blotting against our purified recombinantly expressed NAC (see Figure S1).
